# Bionomics of the oriental latrine fly *Chrysomya megacephala* (Fabricius) (Diptera: Calliphoridae): temporal fluctuation and reproductive potential

**DOI:** 10.1186/s13071-018-2986-2

**Published:** 2018-07-13

**Authors:** Narin Sontigun, Kabkaew L. Sukontason, Tunwadee Klong-klaew, Sangob Sanit, Chutharat Samerjai, Pradya Somboon, Sa-nguansak Thanapornpoonpong, Jens Amendt, Kom Sukontason

**Affiliations:** 1 Graduate School, Chiang Mai University, Chiang Mai, 50200 Thailand; 2 Department of Parasitology, Faculty of Medicine, Chiang Mai University, Chiang Mai, 50200 Thailand; 3 Department of Plant Science and Natural Resources, Faculty of Agriculture, Chiang Mai University, Chiang Mai, 50200 Thailand; 4 Institute of Legal Medicine, Goethe-University Frankfurt, Kennedyallee 104, 60596 Frankfurt am Main, Germany

**Keywords:** *Chrysomya megacephala*, Daily flight activity, Seasonal fluctuation, Reproductive potential, Thailand

## Abstract

**Background:**

*Chrysomya megacephala* is a blow fly species of medical and forensic importance worldwide. Understanding its bionomics is essential for both designing effective fly control programs and its use in forensic investigations.

**Methods:**

The daily flight activity, seasonal abundance related to abiotic factors (temperature, relative humidity and rainfall) and reproductive potential of this species was investigated. Adult flies were sampled twice a month for one year from July 2013 to June 2014 in three different ecotypes (forest area, longan orchard and palm plantation) of Chiang Mai Province, northern Thailand, using semi-automatic funnel traps. One-day tainted beef offal was used as bait.

**Results:**

A total of 88,273 flies were sampled, of which 82,800 flies (93.8%) were caught during the day (from 06:00 to 18:00 h); while 5473 flies (6.2%) were caught at night (from 18:00 to 06:00 h). Concurrently, the abundance of *C. megacephala* was higher in the forest area (*n* = 31,873; 36.1%) and palm plantation (*n* = 31,347; 35.5%), compared to the longan orchard (*n* = 25,053; 28.4%). The number of females was significantly higher than that of males, exhibiting a female to male sex ratio of 2.36:1. Seasonal fluctuation revealed the highest abundance of *C. megacephala* in summer, but low numbers in the rainy season and winter. Fly density was significantly positively correlated with temperature, but negatively correlated with relative humidity. No correlation between numbers of *C. megacephala* with rainfall was found. Activity occurred throughout the daytime with high numbers from 06:00 to 18:00 h in summer and 12:00 to 18:00 h in the rainy season and winter. As for the nocturnal flight activity, a small number of flies were collected in summer and the rainy season, while none were collected in the winter. Dissection of the females indicated that fecundity was highest during the rainy season, followed by winter and summer.

**Conclusions:**

Since the assessment of daily, seasonal activity and the reproductive potential of *C. megacephala* remains a crucial point to be elucidated, this extensive study offers insights into bionomics, which may be considered for integrated fly control strategies and forensic entomology issues.

## Background

The oriental latrine fly, *Chrysomya megacephala* (Fabricius, 1794), is a medically and forensically important blow fly species as its habit and breeding places are within or near to human settlements. Adults are mechanical carriers of a range of pathogens [1–3] and their larvae can cause myiasis in humans and animals [4, 5]. The larvae feed on human corpses, thus they can be beneficial in forensic investigations for estimating the minimum time since death (PMI_min_) [6]. *Chrysomya megacephala* is considered to be the predominant species associated with human corpses in a forensic context in Thailand and Malaysia [7, 8].

Geographically, *C. megacephala* expands worldwide, being present in Asia, Australasia/Oceania, and the Palaearctic, Afrotropical, Nearctic and Neotropical realms [9]. This species is considered to have variable habitats, and in addition to human settlements, have been found across different ecotypes, spanning urban, peri-urban, rural and natural forested areas, and found as high up as 2667 m above sea level [10, 11]. In Thailand, *C. megacephala* was the most abundant blow fly species collected during fly surveys [12, 13] and it was the most important species found on human corpses [7]. Adults are reported as a predominant mechanical carrier of a range of pathogenic bacteria (e.g. *Escherichia coli* O157:H7, *Klebsiella pneumoniae*, *Salmonella typhi*, *Staphylococcus aureus*, *Pseudomonas aeruginosa*) and fungi (e.g. *Aspergillus* spp., *Cladosporium* spp., *Fusarium* spp., *Curvularia* spp.) that can produce diseases such as diarrhea and skin infection in humans [14–16]. Therefore, detailed information on the bionomics of this species is crucial for designing an effective management and improving its consideration in forensic entomology investigations in various environmental habitats. Information such as daily activity, seasonal fluctuation and reproductive potential are vital in this regard.

Several environmental factors, e.g. altitude, rainfall, temperature, relative humidity and land use types can directly affect the distribution and abundance of blow flies [11–13, 17]. In Thailand, the seasonal activity of *C. megacephala* has been investigated in Chiang Mai Province, the northern region [12, 18], and Bangkok Province, the central region [19], while the daily activity pattern of *C. megacephala* has only been reported in Bangkok [20]. Considering that different fly activity can affect their ability as a vector, our aim was to investigate the bionomics of *C. megacephala*, highlighting daily and seasonal activity patterns of the flies in relation to abiotic factors (temperature, relative humidity and rainfall) and ecotypes in Chiang Mai Province, and evaluating the reproductive potential of field collected females.

## Methods

### Study area

**Fig. 1:**
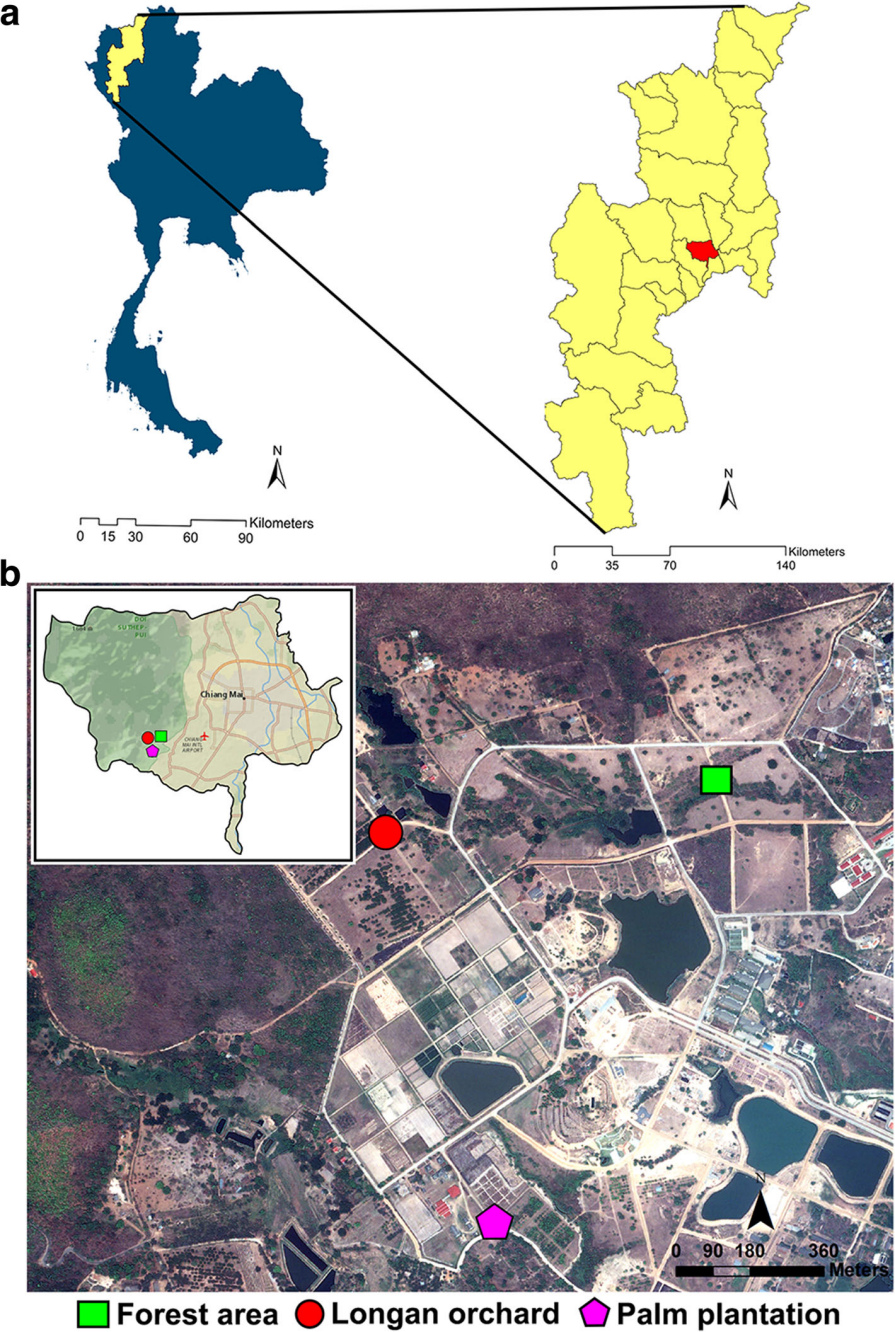
Location of the study area. **a** Map of Thailand showing the study site located in Mueang Chiang Mai District of Chiang Mai Province, northern Thailand. **b** Satellite imagery of the study area comprising three ecotypes: forest area, longan orchard and palm plantation

This study was conducted at Mae Hia Agricultural Research, Demonstrative and Training Center, located in Mueang Chiang Mai District, Chiang Mai Province, northern Thailand (Fig. 1a). Based on their topography, three different ecotypes were chosen as studied sites, comprising a forest area (18°46'01.08"N, 98°56'08.3"E; altitude 344 m), a longan orchard (18°45'56.66"N, 98°55'40.13"E; altitude 374 m) and a palm plantation (18°45'27.841"N, 98°55'48.515"E; altitude 330 m) (Fig. 1b). According to the Thai Meteorological Department report, the climate is divided into three seasons based on rainfall and air temperature values, namely summer (March to May), rainy season (June to October) and winter (November to February).

### Fly collection

Adult fly sampling was performed twice a month for 1 year from July 2013 to June 2014 using semi-automatic funnel traps. The collection procedure was described previously by Klong-klaew et al. [13]. Each trap was baited with 300 g of 1-day tainted beef offal [12], which was put in a plastic container and placed at the base of the trap. Sampling took place for 24 h for each experimental cycle. At each study site, 5 traps were set to operate at 5 different time intervals, respectively, including 06:00 to 09:00 h (early morning), 09:00 to 12:00 h (late morning), 12:00 to 15:00 h (early afternoon), 15:00 to 18:00 h (late afternoon) and 18:00 to 06:00 h (night). These traps were arranged in a straight line, 50 cm apart from each other. After the sampling period of 24 h, the fly net was manually removed from the trap, sealed with masking tape to prevent the flies from escaping, labeled and then transported to the laboratory at Department of Parasitology, Chiang Mai University. During the period of this experiment, hourly temperature (°C) and relative humidity (%) were recorded at each study site on the day of the fly collection using temperature and humidity loggers (EBI 20-TH1; ebro Electronic GmbH & Co. KG, Ingolstadt, Germany). The average monthly air temperature, relative humidity and rainfall from July 2013 to June 2014, and the daily sunrise and sunset times for the experimental periods, were obtained from the Northern Meteorological Center, situated in Chiang Mai International Airport (18°46'16.8"N, 98°59'00.1"E; altitude 314 m), Mueang Chiang Mai District, Chiang Mai Province.

### Fly identification

In the laboratory, all trapped flies were frozen at -20 °C for 2 h. They were then identified individually into species under a dissecting microscope (Olympus, Tokyo, Japan) using the taxonomic identification key of Kurahashi & Bunchu [21]. Only *C. megacephala* was sexed, counted and analyzed in this study.

### Reproductive status and fecundity of *C. megacephala* females

**Fig. 2:**
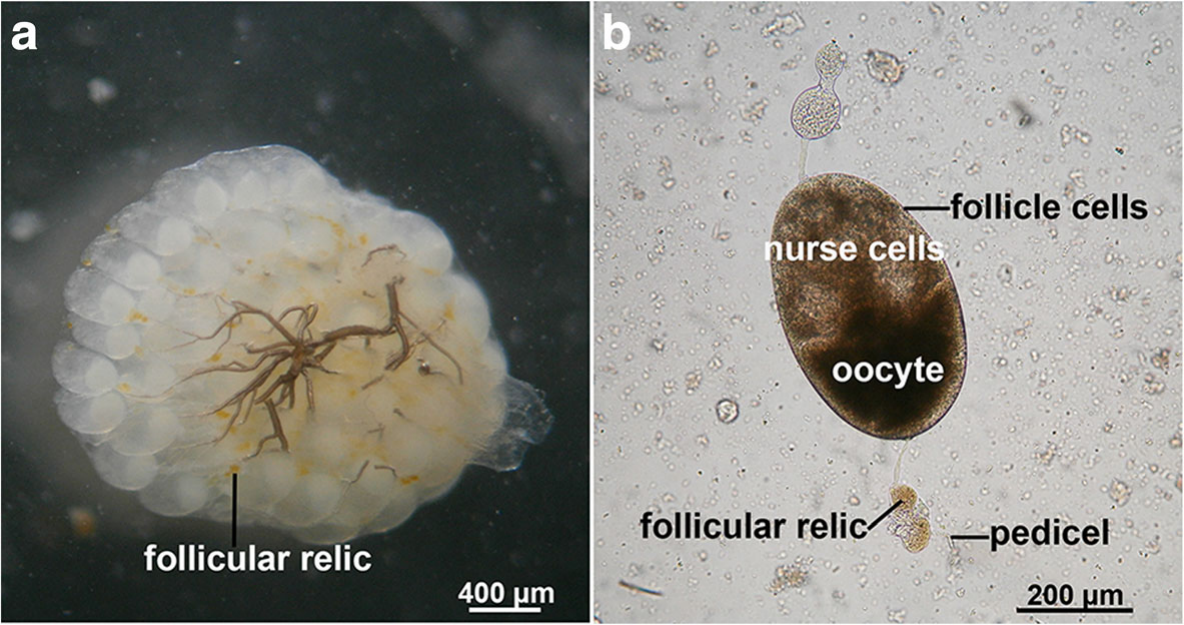
Photomicrograph of yellow follicular relics from a parous female. **a** Ovary contains bright yellow follicular relics in the pedicels at the base of the ovarioles. **b** Morphological features of ovariole with yellow follicular relic in the pedicel

The ovaries of *C. megacephala* were individually dissected on a glass slide in phosphate buffer saline (pH 7.4) under a dissecting microscope (Olympus) to determine ovarian status (non-gravid or gravid) and parity status (nulliparous or parous) for the differentiation between young and old females. If the number of captured specimens were greater than 100, 100 females per trap were randomly selected. Conversely, all females were dissected if the number of captured females were equal to or less than 100. Flies were classified as gravid if the ovaries contained mature eggs (stage VIII), while those with ovaries of a younger stage (I-VII) were categorized as non-gravid, based on the criteria previously described [22]. The parity was categorized as parous (females that have ever laid eggs) or nulliparous (females that have never laid eggs) by the presence or absence of yellow follicular relics (the remnants of the nurse cells and follicular epithelium which are retained in the ovarioles after oviposition) in the pedicel at the base of ovarioles (Fig. 2) [23]. When females have follicular relics in the pedicels of their ovarioles they were classified as parous; females lacking these follicular relics were classified as nulliparous. Additionally, the number of ovarioles big enough to count, and/or the number of mature eggs in their ovaries were counted and recorded.

### Statistical analysis

To normalize the abundance, *C. megacephala* numbers (x) were transformed into log_10_ (x + 1) prior to statistical analysis. Since only small numbers of flies were collected during the night, the data were analyzed separately between day traps (diurnal flight activity: from 06:00 to 18:00 h) and night traps (nocturnal flight activity: from 18:00 to 06:00 h). The effects of temperature and relative humidity on trap catches during the daytime were assessed using one-way analysis of variance (ANOVA), followed by a Games-Howell *post-hoc* test. For the patterns of diurnal flight activity, one-way ANOVA and Dunnett’s T3 *post-hoc* test was used to compare differences in the average number of flies caught between time of day (06:00 to 09:00 h, 09:00 to 12:00 h, 12:00 to 15:00 h, and 15:00 to 18:00 h), using the combined number of flies caught at each study site. Spearman’s rank correlation coefficient (*r*_*s*_) was used to determine the influence of climatic factors (temperature, relative humidity and rainfall) on fly density and fecundity. Differences in the average number of ovarioles between seasons and study sites were tested using one-way ANOVA and Games-Howell *post-hoc* tests. Mann-Whitney U-test was used to compare the abundance of sexes (males and females) and the reproductive status of females, i.e. ovarian status (gravid and non-gravid) and parity status (parous and nulliparous). Pearson’s Chi-square test was performed to assess the correlation between the parity status of females and seasons and study sites. Statistical analyses were performed using SPSS software version 17.0 for Windows (SPSS Inc., Chicago, Illinois, USA). All statistical tests were considered significant at the *P* < 0.05 level.

## Results

### Abundance of *C. megacephala*

Altogether 88,273 *C. megacephala* were sampled during day and night at all three study sites. The highest number of *C. megacephala* were captured in the forest area (*n* = 31,873; 36.1%), followed by the palm plantation (*n* = 31,347; 35.5%) and longan orchard (*n* = 25,053; 28.4%). During daytime many more (*n* = 82,800; 93.8%) specimens were sampled than during night (*n* = 5473; 6.2%).

### Seasonal diurnal activity of *C. megacephala*

**Fig. 3:**
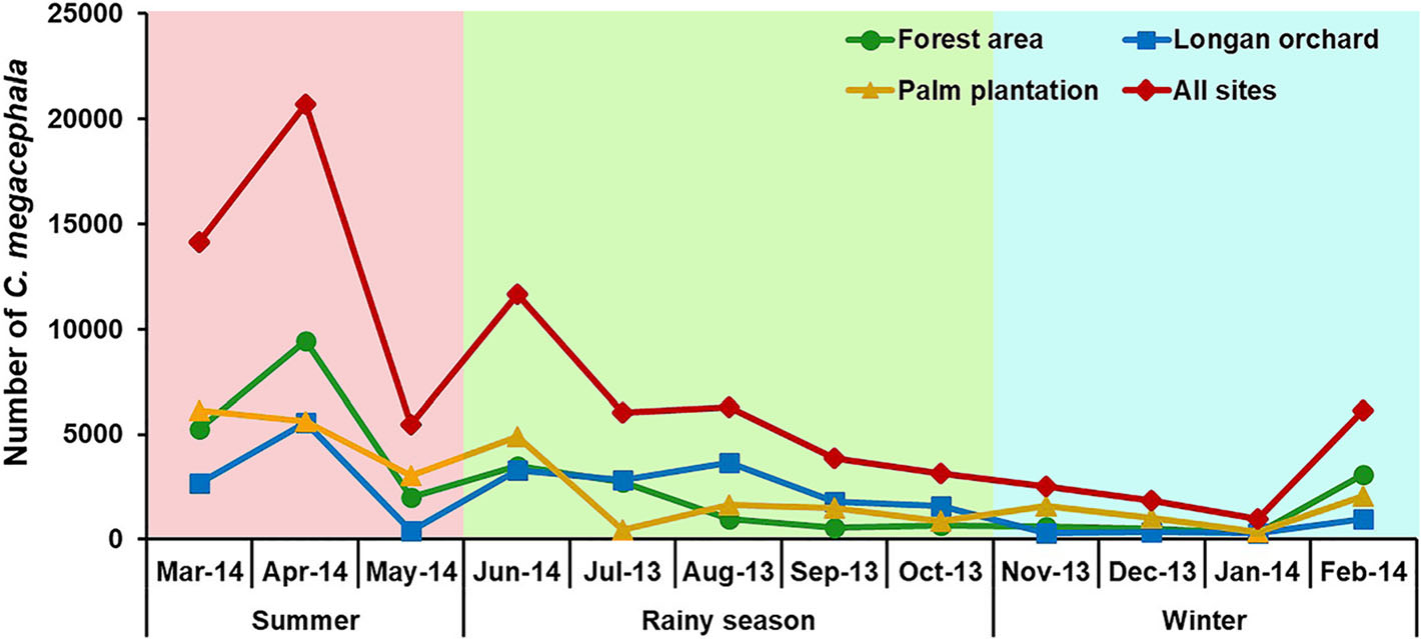
Seasonal fluctuation in population density of *C. megacephala* collected by day traps from July 2013 to June 2014

To assess the overall seasonal changes in abundance of *C. megacephala*, data from each study site were combined. *Chrysomya megacephala* was perennial in the studied sites (Fig. 3). The seasonal trend of the total number captured in these study areas showed that high numbers of flies were trapped in the summer with a peak in April 2014, before populations decreased rapidly at the end of summer (May 2014), rising again in the early rainy season (June 2014), and eventually declined through the rainy season and winter. During the sampling period, peak numbers of flies were found in April 2014 (*n* = 20,673), while being lowest in January 2014 (*n* = 962). A similar trend was noticed in all three study sites (Fig. 3).

The abundance of females (*n* = 58,188) was significantly higher than males (*n* = 24,612) at all three study sites (Mann-Whitney U*-*test: *U* = 24362.500, *Z* = -8.571, *P* < 0.0001), with an overall female to male sex ratio of 2.36:1.

### Influence of climatic factors and ecotypes on fly density

**Fig. 4:**
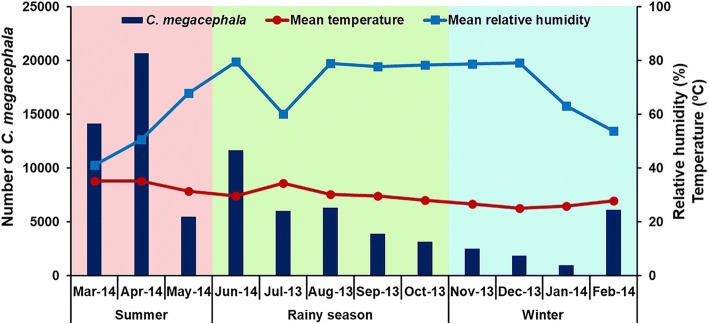
Fluctuation of the overall *C. megacephala* population density relative to temperature and relative humidity recorded at the study sites from July 2013 to June 2014

**Fig. 5:**
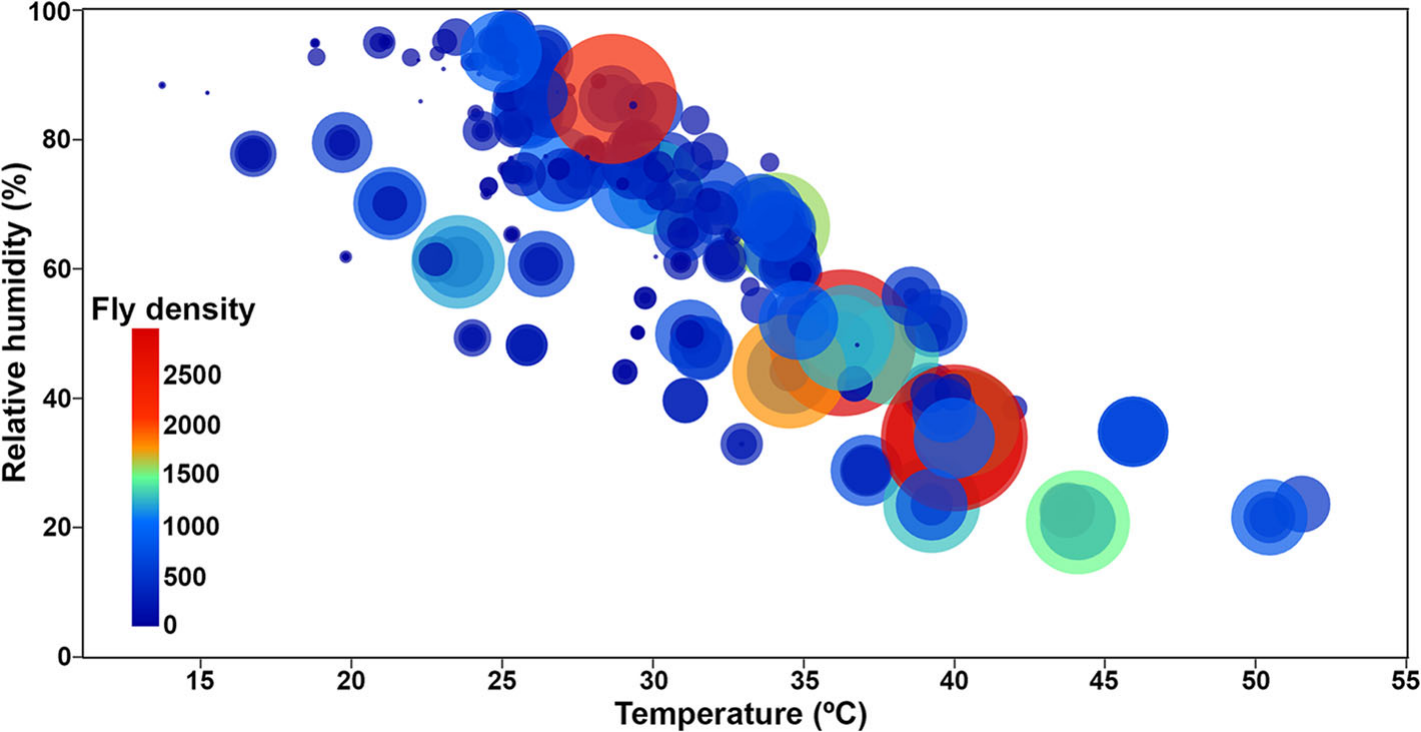
The number of captured *C. megacephala* in relation to study site temperature (°C) and relative humidity (%). Each bubble shows fly density in each temperature and relative humidity recorded in each trap catch; a bigger size of bubble shows a greater number of captured flies

Spearman’s correlation analysis showed that overall, *C. megacephala* abundance was moderately positively correlated with temperature (Spearman’s correlation: *r*_*s*_ = 0.476, *P* < 0.0001), but moderately negatively correlated with relative humidity (Spearman’s correlation: *r*_*s*_ = -0.411, *P* < 0.0001) (Table 1, Fig. 4). The correlation between the climatic factors of each study site and its related fly abundance is shown in Table 1. In all three study sites, fly population showed a positive correlation with temperature, but a negative correlation with relative humidity. During the sampling period, *C. megacephala* was sampled at temperatures between 13.7–51.5 °C, and a relative humidity between 20.8–96.8% (Fig. 5). Statistical analysis revealed that the highest abundance occurred at temperatures > 35 °C and relative humidity < 50% (Game-Howell, *P* < 0.05), while at temperatures < 25 °C and relative humidity > 70% the abundance was lowest (Game-Howell, *P* < 0.05) (Table 2).

**Table 1:** Spearman’s rank correlation coefficient values of climatic factors and fly density of *C. megacephala*

		Study sites			
		Forest area	Longan orchard	Palm plantation	All sites
Temperature	*r*_*s*_	0.507	0.468	0.444	0.476
	*P*	< 0.0001	< 0.0001	< 0.0001	< 0.0001
Relative humidity	*r*_*s*_	-0.545	-0.289	-0.382	-0.411
	*P*	< 0.0001	0.004	< 0.0001	< 0.0001

**Table 2:** Abundance of *C. megacephala* collected by day traps in each range of temperature and relative humidity. Different letters indicate significant differences (Game-Howell *post-hoc* test, *P* < 0.05)

Variable	*n*	Number of flies*
Temperature (°C)		
< 25	57	1.39 ± 0.92^a^
25–30	99	1.88 ± 0.65^b^
30–35	81	2.12 ± 0.73^b^
> 35	51	2.57 ± 0.54^c^
One-way ANOVA		*F*_(3, 284)_ = 26.034, *P* < 0.0001
Relative humidity (%)		
< 50	64	2.49 ± 0.65^a^
50–70	68	2.06 ± 0.70^b^
> 70	156	1.72 ± 0.80^c^
One-way ANOVA		*F*_(2, 285)_ = 24.458, *P* < 0.0001

*Abbreviation*: *n*, the number of samplings falling into each range of temperature and relative humidity obtained in a one-year study

*Mean of log_10_ (x + 1) ± SD

Spearman’s correlation analysis revealed a correlation of fly density and weather station temperature, relative humidity and actual rainfall. There was a moderate positive correlation between fly density and temperature (Spearman’s correlation: *r*_*s*_ = 0.444, *P* < 0.0001), while there was a weakly negative correlation with relative humidity (Spearman’s correlation: *r*_*s*_ = -0.307, *P* < 0.0001). There was no correlation between fly density and actual rainfall (Spearman’s correlation: *r*_*s*_ = -0.049, *P* = 0.404).

### Daily flight activity of *C. megacephala*

**Fig. 6:**
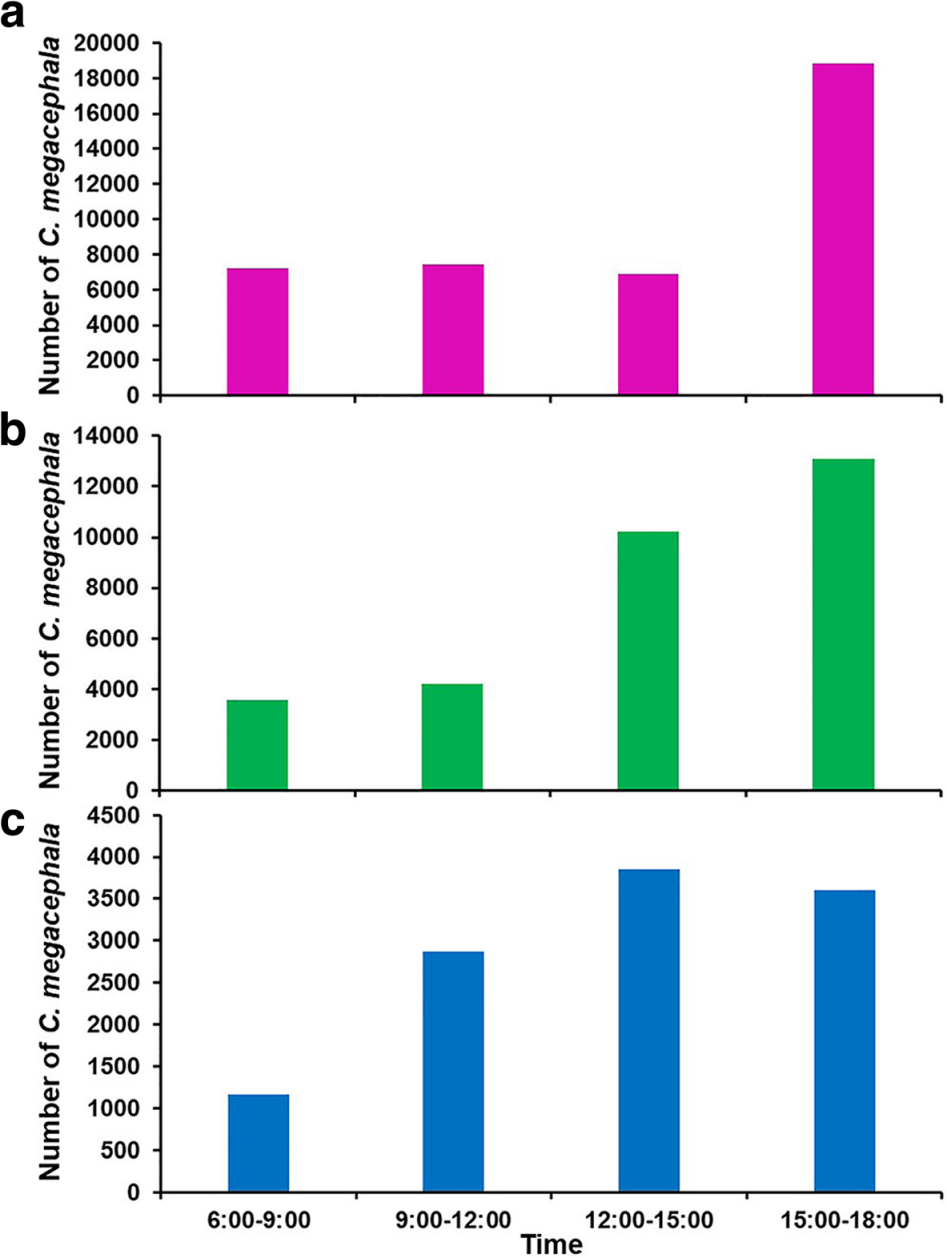
Diurnal flight activity of the overall *C. megacephala* population density in summer (**a**), rainy season (**b**) and winter (**c**) from July 2013 to June 2014

A total of 82,800 flies were collected by day traps (06:00 to 18:00 h) with most of the specimens collected in the afternoon period from 12:00 to 18:00 h (68.1% of the total caught) (Table 3). This pattern of diurnal activity was different between seasons (Table 3, Fig. 6). In summer (Fig. 6a), although high numbers of flies were caught between 15:00 to 18:00 h, the average numbers captured during the intervals were not significantly different (ANOVA: *F*_(3, 32)_ = 1.616, *P* = 0.205). In the rainy season (Fig. 6b) and winter (Fig. 6c), the average fly numbers captured from 12:00 to 15:00 h and 15:00 to 18:00 h (Dunnett T3, *P* > 0.05) were significantly higher than those captured from 06:00 to 09:00 h and 09:00 to 12:00 h (Dunnett T3, *P* < 0.05).

**Table 3:** Number of *C. megacephala* collected by day traps. Different letters in the same column indicate significant differences (Dunnett T3 *post-hoc* test, *P* < 0.05)

Sampling time periods, h	All seasons	Season		
		Summer	Rainy season	Winter
6:00–9:00	2.08 ± 0.78^a^	2.81 ± 0.31^a^	2.13 ± 0.51^a^	1.48 ± 0.84^a^
9:00–12:00	2.31 ± 0.53^ab^	2.69 ± 0.57^a^	2.27 ± 0.42^a^	2.07 ± 0.52^ab^
12:00–15:00	2.65 ± 0.36^b^	2.77 ± 0.42^a^	2.77 ± 0.25^b^	2.40 ± 0.32^b^
15:00–18:00	2.65 ± 0.62^b^	3.14 ± 0.53^a^	2.61 ± 0.69^ab^	2.35 ± 0.36^b^
One-way ANOVA	*F*_(3, 140)_ = 7.818, *P* < 0.0001	*F*_(3, 32)_ = 1.616, *P* = 0.205	*F*_(3, 56)_ = 5.317, *P* = 0.003	*F*_(3, 44)_ = 6.952, *P* = 0.001

Data are presented as mean of log_10_ (x + 1) ± SD for each time interval obtained from monthly data from each of three study sites in a one-year study

**Fig. 7:**
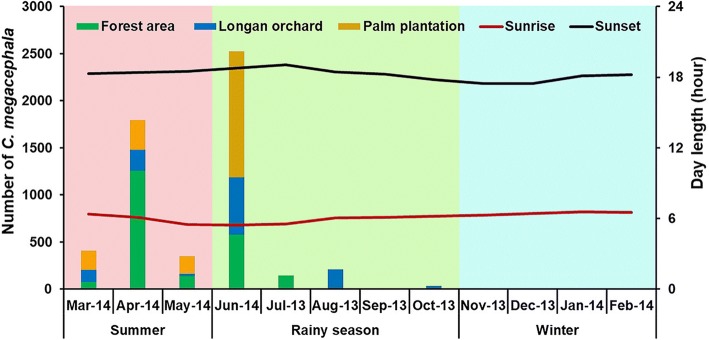
Seasonal density of *C. megacephala* collected by night traps from July 2013 to June 2014

Few *C. megacephala* (*n* = 5473) were collected at night (18:00 to 06:00 h). Small numbers were captured in the summer and rainy season when sunset was after 18:00 h and sunrise before 06:00 h. No flies were captured in winter while sunset was before 18:00 h and sunrise after 06:00 h (Fig. 7).

### Fecundity and reproductive status of *C. megacephala* females

**Fig. 8:**
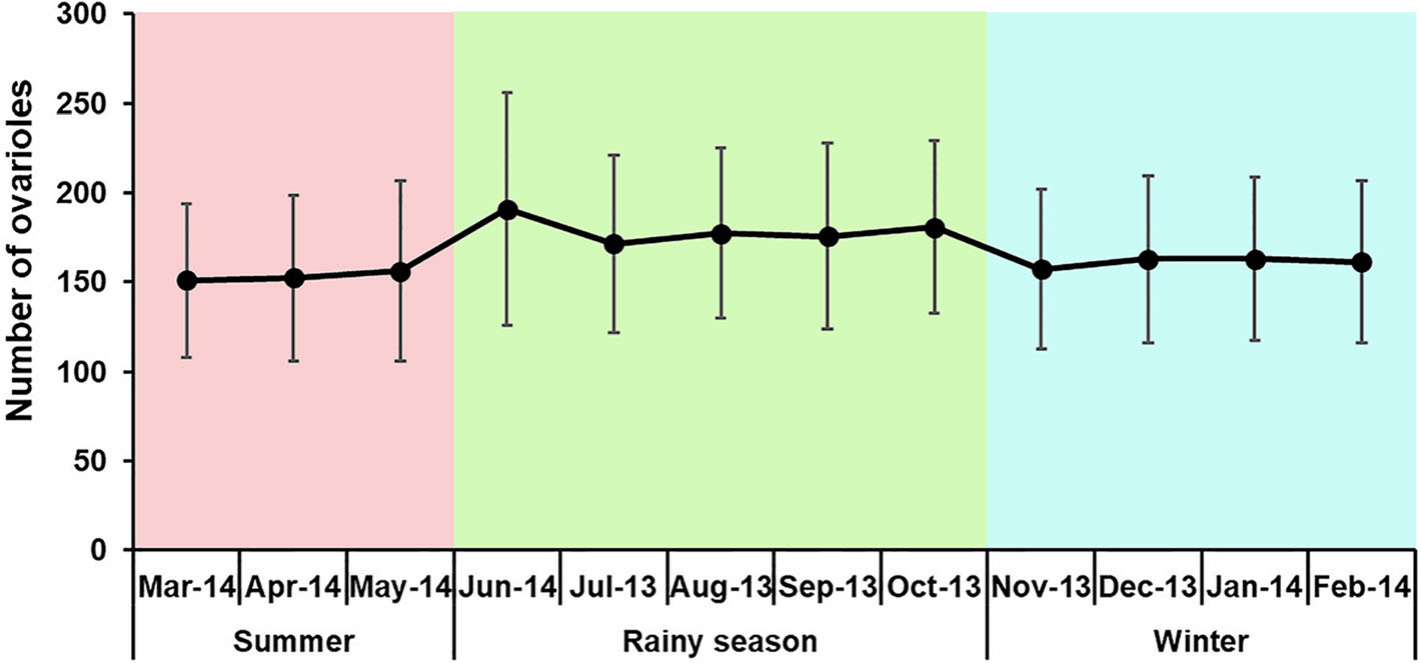
Seasonal variation of fecundity of *C. megacephala* females collected from July 2013 to June 2014. Data are presented as mean ± SD

Analysis of seasonal fecundity showed that the highest and lowest numbers of ovarioles was 190.9 (June 2014) and 151.0 (March 2014), respectively (Fig. 8). The number of ovarioles statistically differed among seasons, with the average number in the rainy season being significantly higher than in winter and summer (Game-Howell, *P* < 0.05) (Table 4). No significant differences in ovariole quantity were observed between the three study sites (ANOVA: *F*_(2, 2375)_ = 0.499, *P* = 0.607). Spearman’s correlation analysis showed that the number of ovarioles obtained from all three study sites was not correlated with temperature (Spearman’s correlation: *r*_*s*_ = -0.027, *P* = 0.188), and weakly positively correlated with relative humidity (Spearman’s correlation: *r*_*s*_ = 0.131, *P* < 0.0001).

**Table 4:** Fecundity and parity status of *C. megacephala* females in each season. Different letters indicate significant differences (Game-Howell *post-hoc* test, *P* < 0.05)

Season	Fecundity		Parity status		
	No. of females counted	No. of ovarioles (mean ± SD)	Nulliparous, *n* (%)	Parous, *n* (%)	Total, *n* (%)
Summer	673	154.5 ± 45.8^a^	3853 (50.8)	3731 (49.2)	7584 (100)
Rainy season	1036	178.2 ± 54.1^b^	5410 (57.6)	3977 (42.4)	9387 (100)
Winter	669	163.7 ± 46.0^c^	2486 (60.7)	1608 (39.3)	4094 (100)
Total	2378	*F*_(2, 2375)_ = 49.214, *P* < 0.0001	11,749 (55.8)	9316 (44.2)	21,065 (100)

**Fig. 9:**
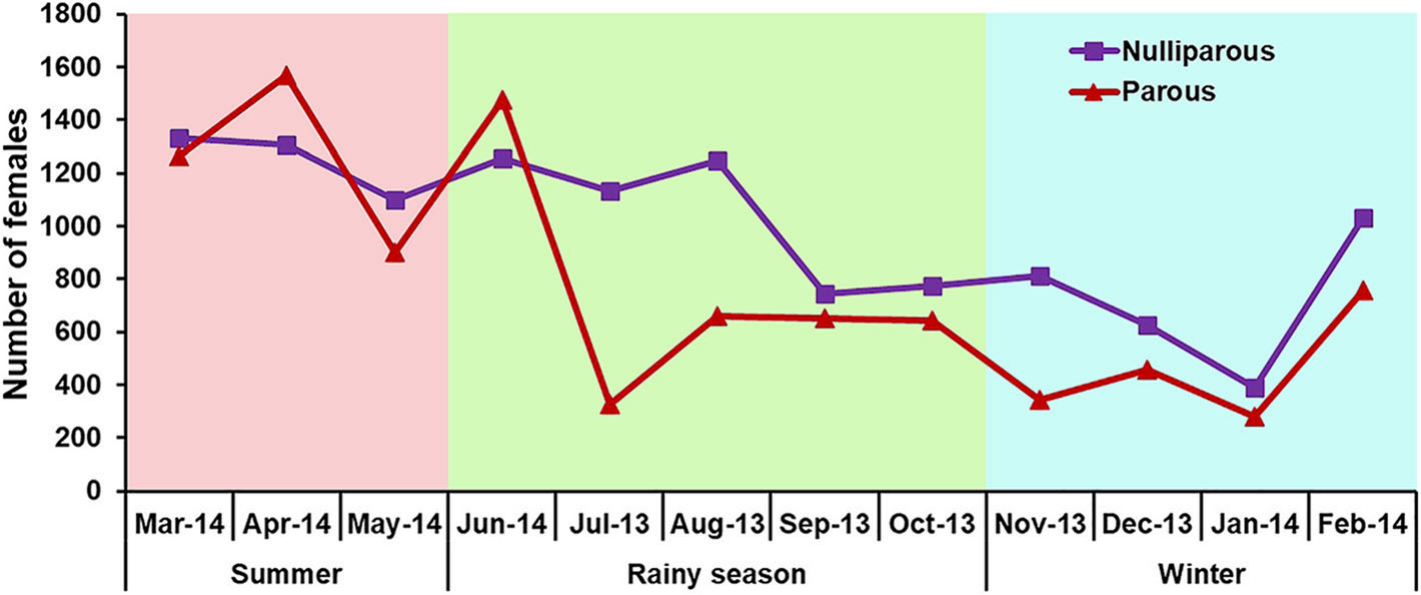
Seasonal variation of parity status of *C. megacephala* females collected from July 2013 to June 2014

Of the total 21,065 female *C. megacephala* dissected, 49.4% were non-gravid and nulliparous, 42.2% non-gravid and parous, 6.3% gravid and nulliparous and 2.1% gravid and parous. Non-gravid females (*n* = 19,299; 91.6%) were significantly higher in numbers than gravid specimens (*n* = 1766; 8.4%) (Mann-Whitney U*-*test: *U* = 22342.500, *Z* = -15.363, *P* < 0.0001). When analyzing the parity status of the flies, 55.8% (*n* = 11,749) were nulliparous and therefore occurred significantly more frequently than parous females (*n* = 9316; 44.2%) (Mann-Whitney U-test: *U* = 54523.000, *Z* = -3.692, *P* < 0.0001). The seasonal fluctuation of parity status is shown in Fig. 9. The number of nulliparous and parous obtained from all three study sites showed that there were more nulliparous than parous females throughout the year, except in the months April and June 2014, which showed higher numbers of parous than nulliparous flies (Fig. 9). The parity status was significantly correlated with season (Pearson’s Chi-square test: *χ*^2^ = 129.737, *df* = 2, *P* < 0.0001). Nulliparous flies were proportionally more abundant than parous in all seasons (Table 4). However, no significant correlation of the parity status with study site was shown (Pearson’s Chi-square test: *χ*^2^ = 0.752, *df* = 2, *P* = 0.687).

## Discussion

Understanding the bionomics of blow flies is imperative as one of the key pieces of information to be integrated in fly control strategies, if targeted at the right place, at the right time, with the right approach. Based on the sampling of 88,273 specimens, this work presents significant data on *C. megacephala*, highlighting comprehensive daily and seasonal activity year-round in three different ecotypes, correlating fly density with various abiotic factors, and evaluating the reproductive potential of field-collected specimens.

*Chrysomya megacephala* had the highest abundance in summer, peaking in April 2014, and gradually declined during rainy season and winter. Similar findings were reported in earlier studies in Thailand [12, 18]. By comparison, Phasuk et al. [19] showed that *C. megacephala* had trimodal peaks, with the highest abundance in January, followed by September and June. Seasonal abundance of blow flies studied in India by Wall et al. [24] found that population of *C. megacephala* increased at the beginning of rainy season, and then declined in the dry hot season. In Brazil, the greatest constant abundance of *C. megacephala* occurred between October and February (end of spring and all of summer) and reached the highest population peak in December (highest temperatures of the year) [17, 25]. Such information indicated that fluctuation in *C. megacephala* abundance is specific to the location and the population changes according to season. In this regard, investigation on seasonal activity should be carried out in several regions for effective fly control strategy in that specific area.

Higher numbers of trapped females than males suggest that females were active in searching for a protein food source, oviposition, and/or breeding places, while males are not attracted to protein sources for physiological requirements, but for mating needs [26–28]. Similar findings were shown in other studies, collecting more females from meat-baited trap than males [12, 18, 19, 24].

Blow fly density was significantly influenced by climatic factors. Our results clearly showed that *C. megacephala* density is positively correlated with temperature and negatively correlated with relative humidity. Such results indicated that *C. megacephala* density increased when the temperature was high and the relative humidity was low, as the highest abundance was observed in summer when the temperature was high and the relative humidity low. In contrast, *C. megacephala* abundance was low in the rainy season and winter when the temperature was low and relative humidity high. Therefore, variations in the temperature and relative humidity had significant effects on the abundance and seasonal fluctuation of *C. megacephala* in this studied area. This finding is in agreement with the previous report on the same species in Thailand by Ngoen-klan et al. [12]. However, it is in contrast with the finding of Phasuk et al. [19] which showed a negative correlation of fly density with temperature and relative humidity. In India, *C. megacephala* abundance was positively associated with relative humidity, but negatively associated with temperature and rainfall [24]. In Brazil, *C. megacephala* density was positively related to temperature and rainfall [17]. In addition to temperature and relative humidity, our results for the north of Thailand clearly show that the abundance of *C. megacephala* is not correlated with rainfall, which is in contrast with findings in India [24] and Brazil [17, 25].

Among environmental factors, temperature has been considered essential as a factor in blow fly biology since it can directly impact the development, survival, longevity, behavior and population dynamics of flies [12, 29–31]. In the current study, *C. megacephala* was trapped between 13.7 and 51.5 °C, with the highest number of flies occurring at temperatures > 35 °C. Such results indicate that *C. megacephala* is able to adapt to a broad range of temperatures, with adult activity most intense at high temperature and reduced at lower temperatures. This is in agreement with detailed studies by Ngoen-klan et al. [12] who found *C. megacephala* at temperatures ranging from 15 to 40 °C, with a high peak in the late summer (high temperature) and low peak in the rainy season and winter (low temperature). In addition, Sukontason et al. [29] revealed that *C. megacephala* larvae developed rapidly in April (31.4 °C) and grew slowly in the rainy season and winter. Other studies also showed that *C. megacephala* developed more rapidly with increasing temperatures, but developed slowly at low temperatures [30–32].

Blow flies are considered to be diurnal insects and inactive at night [20, 33, 34]. However, some studies found nocturnal activity and even oviposition in blow flies [35–37]. In the current study, *C. megacephala* exhibited a diurnal activity, with a peak in the afternoon (12:00 to 18:00 h). However, the small-scale diurnal activity pattern of flies regarding the time of the day differs across seasons. Minimal numbers of *C. megacephala* were captured during nighttime. This is in accordance with Nazni et al. [38], who also demonstrated that *C. megacephala* exhibited both diurnal and nocturnal activities, but much higher numbers of flies in daytime traps than nighttime traps. Conversely, Sucharit & Tumrasavin [20] stated an activity peak of *C. megacephala* at 16:00 to 18:00 h, and no flies during the night, as well as Soares & Vasconcelos [39], who observed neither nocturnal activity nor oviposition during nighttime. In the present study, minimal numbers of flies were captured during nighttime in summer and rainy season as the sunset was after 18:00 h (nighttime trap was set from 18:00 to 06:00 h), thus flies were still able to be active, entering the traps. In contrast, no flies were captured in the night in the winter as the sunset was before 18:00 h. Such results suggest that light might stimulate fly activity [40, 41], as reported by Sucharit & Tumrasavin [20], who found that no flies were collected after sunset.

Fecundity plays an important role in population dynamics since it determines the population growth potential [42]. In the present study, we found fecundity was significant differences between seasons; however, fecundity showed no relationship with temperature and just a very weak positive correlation with relative humidity. Similar findings were observed for *C. megacephala* [43], and other blow fly species like *Chrysomya albiceps* [44] and *Lucilia eximia* [45], which fecundity was not significantly affected by changes in temperature. This result suggested that fecundity is probably influenced by the ecological niches occupied by this species, which seasonal and environmental changes may affect the abundance of food resources, e.g. the presence of plants, fruits, animal carcasses or dung. Many studies have revealed that the larval nutrition plays a key role in life-history characters in insects such as growth, survival, body size, longevity and reproduction [46–49]. The fitness of a female fly is determined by the number of offspring that she can produce, which depends on her body size. Study on the relationship between fecundity and body size of three blow fly species (*C. megacephala, C. albiceps* and *L. eximia*) in Brazil revealed that fecundity was positively correlated with body size [43–45], indicating that fecundity increased when the body size was large.

Determination of the age structure of adult blow flies in the field is essential for understanding their ecology and behavior which vary with age [27, 50–52]. Our results showed that many more females were non-gravid than gravid, and more nulliparous than parous. This finding is consistent with those of Spradbery [27] and Davies [52]. This should be considered when planning a sound fly control program, which e.g. applying targeted control measures before the population reaches peaks of gravidity and reproduction. From a forensic point of view, it is of interest that gravid blow fly females are much more attracted by stages of initial decay, while non-gravid flies were much more present during advanced decay [51]. Different ages affect the behavior of blow flies, e.g. as non-gravids required a protein food for egg maturation, while gravid specimens required protein food for egg deposition. In the present study, trapped females showed a marked bias in ovarian status, females were mostly non-gravid, suggesting that meat-baited traps act as a protein source rather than as an oviposition site.

As for the parity determination, most non-gravid dissected flies from all sites were nulliparous, indicating the newly emerged young fly populations. Conversely, the presence of follicular relics in the ovaries provides a rough estimate of the female age; therefore, parous females are only classified as old flies. The majority of dissected females were nulliparous rather than parous, indicating much younger flies than older in the studied areas. Such result suggested trap bias in parity status of females. This was probably due to the greater protein requirements to begin ovarian development and mating needs in nulliparous females compared to parous females, which had previous exposure to protein and were mated. As previously reported in *L. sericata* [53], females that had recently oviposited showed a lower attractive response to liver odor than protein-deprived or fully gravid females. Moreover, parity status appears to be related to seasons, suggesting that seasonality affects reproductive activity of *C. megacephala* females*.*

## Conclusions

Our results demonstrated that temperature and relative humidity significantly influenced the abundance of *C. megacephala*. *Chrysomya megacephala* was found year-round, with maximum abundance in summer. Furthermore, the patterns of diurnal flight activity differed between seasons and time of day, with peak activity occurring from 06:00 to 18:00 h in summer and 12:00 to 18:00 h in the rainy season and winter. Our findings provide an insight into essential bionomics, which can be useful for designing effective fly control strategies, in terms of when to implement control programs. Moreover, the results regarding the reproductive potential and age structure of females obtained in this study might also help to predict fly activity and oviposition in a forensic context, helping to evaluate the time since death.

## Abbreviations

PMI_min_: Minimum time since death
ANOVA: Analysis of variance
SPSS: Statistical Package for the Social Sciences
